# The validity of visual and hearing impairment in predicting dementia and cognitive impairment in older adults: a systematic review and meta-analysis

**DOI:** 10.3389/fnagi.2025.1656686

**Published:** 2026-01-16

**Authors:** Qingwen Gan, Yiling Yuan, Qianqian Hu, Yonghui Zhang

**Affiliations:** 1 Jiangxi Mental Hospital and Affiliated Mental Hospital of Nanchang University, Nanchang, China; 2 Jiangxi Medical College, Shangrao, China; 3 School of Nursing, Nanchang University, Nanchang, China

**Keywords:** cognitive impairment, dementia, dual sensory impairment, hearing impairment, visual impairment

## Abstract

**Objective:**

The number of people with dementia and cognitive impairment is rising every year as the older population grows. The higher prevalence and mortality rates of dementia and cognitive impairment place an enormous burden on healthcare and economic systems worldwide. Studies have shown that older adults with sensory impairments are at a higher risk of developing dementia and cognitive impairment than normal older adults. There is a lack of systematic reviews on the relationship between sensory impairment and dementia and cognitive impairment in older persons. This meta-analysis aimed to analyze the correlation of visual impairment, hearing impairment, and dual sensory impairment with dementia and cognitive impairment, and to provide guidance for reducing the incidence of dementia and cognitive impairment in older adults.

**Methods:**

Computerized searches were conducted using the CNKI, Wanfang, Vip, Sinomed, PubMed, Web of Science, Cochrane Library, and Embase databases. Supplementary searches were performed on 2 clinical trial registries.

**Results:**

Meta-analysis was performed by log-transforming the study-specific estimates. The heterogeneity of studies was characterized by Q-test and *I*^2^. The results of the studies indicated that dual sensory impairment was associated with dementia [OR 95% CI 1.66 (1.47, 1.86)], visual impairment was associated with dementia [OR 95% CI 1.60 (1.48, 1.74)], hearing impairment was associated with dementia [OR 95% CI 1.26 (1.22, 1.31)], dual sensory impairment was associated with cognitive impairment [OR 95% CI 2.08 (1.70, 2.54)], visual impairment was associated with cognitive impairment [OR 95% CI 1.84 (1.44, 2.36)], and hearing impairment was associated with cognitive impairment in old age [OR 95% CI 1.50 (1.36, 1.65)].

**Conclusion:**

Sensory impairment is a predictor of dementia and cognitive impairment. Healthcare professionals should prioritize screening older adults with sensory impairments to reduce the incidence of dementia and cognitive impairment.

**Systematic review registration:**

https://www.crd.york.ac.uk/prospero/, identifier CRD42024606342.

## 1 Introduction

Dementia is defined as a decline in one or more cognitive domains (memory, language, executive function, complex attention, and perceptual-motor) that interferes with the independence of the person’s daily activities. Research indicates that with advancing age, particularly in older adults, cortical atrophy, increased neuronal apoptosis, and reduced neurotransmitter secretion lead to a physiological decline in cognitive function (Liu et al., 2023). This provides the pathological basis for the onset of dementia, which is also a major cause of disability and mortality among older adults (Liu et al., 2023). Studies have reported that dementia currently affects 55 million people worldwide, and the number of people with dementia is expected to reach 152 million by 2025 (World Health Organization [WHO], 2022; Liu et al., 2023). The number of people with dementia is rising every year as older adults grow, and its financial burden is increasing, with the cost of dementia care expected to exceed $2.8 trillion by 2030 (World Health Organization [WHO], 2022; Shang et al., 2021). Its high prevalence and mortality rates place a huge burden on global healthcare and economic systems. Mild cognitive impairment is a state of reversibility in which the degree of impairment of a variety of cognitive functions, such as memory, attention, and executive function, exceeds the range of impairments brought about by normal aging with age in an individual, but has not yet reached the diagnostic range of dementia, and is an intermediate state between normal cognition and dementia (Sohn et al., 2023). Cognitive dysfunction is the fifth leading cause of disability in older adults and imposes a huge physical, psychological, and economic burden on the patient as well as on the family, caregivers, and society (Vu et al., 2021). It is estimated that by 2050 there will be more than 2 billion older adults and the number of patients with cognitive impairment will increase twofold (Vu et al., 2021). Therefore, it is particularly important to identify modifiable risk factors for dementia and cognitive impairment in older adults in time and to implement interventions to slow disease progression.

Sensory impairments, including visual impairment, hearing impairment, and dual sensory impairment (visual impairment and hearing impairment), seriously affect the quality of life of older adults (Yang et al., 2024). Visual impairment is an age-related condition that is the third leading cause of disability in older adults (Vu et al., 2021). Previous studies have shown that visual impairment is closely related to neurons or microvessels in the brain of patients with dementia or cognitive impairment, and is one of the early symptoms of dementia (Vu et al., 2021). Hearing impairment is the most common chronic sensory disorder in older adults; approximately 50% suffer from disabling hearing impairment (Xu et al., 2024). The Lancet Dementia Council reported hearing impairment as a major risk factor for dementia (Livingston et al., 2020). Older adults with dual sensory impairment have a higher risk of cognitive impairment than older adults with single sensory impairment (Yang et al., 2024). Studies have reported that sensory impairment increases the cognitive load of the patient, leading to a decrease in physical activity and social interaction, which in turn can increase the risk of cognitive decline and dementia in older adults (Yang et al., 2024).

Currently, studies have shown that older adults with sensory impairments are at higher risk of developing dementia and cognitive impairment than normal older adults (Byeon et al., 2021; Ge et al., 2021; Huang et al., 2023). However, a study also reported results significantly different from theirs (Hong et al., 2016). This may be due to the inconsistent results of the studies due to differences in sample size, follow-up time, and measurement methods. Currently, most existing studies focus singularly on the correlation between hearing impairment, visual impairment, or dual sensory impairment and dementia or cognitive impairment, with fewer systematic evaluations of the three sensory impairments about dementia and cognitive impairment. Therefore, this study aimed to comprehensively and systematically evaluate the correlation of hearing impairment, visual impairment, and dual sensory impairment with dementia and cognitive dysfunction in older adults to identify the predictors of their disease progression and to provide guidance for interventions to delay dementia and cognitive impairment in older adults.

## 2 Materials and methods

This systematic review strictly follows the Preferred Reporting Items for Systematic Reviews and Meta-Analyses (PRISMA) statements. This systematic review has been registered with the international prospective systematic review registration platform PROSPERO (CRD42024606342).

### 2.1 Search strategy

2 researchers systematically searched the China National Knowledge Infrastructure (CNKI), Wanfang, Vip, Sinomed, PubMed, Web of Science, the Cochrane Library, and Embase database for literature on hearing impairment, visual impairment, and dual sensory impairment related to dementia and cognitive impairment in older adults. The references of the included studies were also traced back to obtain a more comprehensive literature review. Supplementary searches were conducted for relevant studies that had not yet been published in two clinical trial registries (Clinical Trials.gov and the Chinese Clinical Trial Registry). The search was conducted from the time of library construction to January 2025. The search was conducted using subject terms plus free words, and the main search strategies were as follows: (“visual acuity” OR “vision acuity” OR “visual impairment” OR “vision impairment” OR “visual loss” OR “vision loss” OR “partial sight” OR “blindness” OR “hearing acuity” OR “hearing Loss” OR “hearing impairment” OR “hearing disorders” OR “hearing difficulty” OR “dual sensory loss” OR “dual sensory impairment” OR “dual impairments” OR “sensory impairments”) AND (older* OR elder* OR senior* OR geriatric*) AND (cogniti* OR alzheimer* OR dementia*). Specific search strategies for each database are described in the Supplementary materials.

### 2.2 Inclusion exclusion criteria

The inclusion criteria were developed according to the PRISMA.

The inclusion criteria for the study were as follows:

(1) study participants were older adults ≥ 60 years of age with hearing impairment, visual impairment, or dual sensory impairment;

(2) the outcome indicator was dementia or cognitive impairment;

(3) the type of study was cross-sectional, cohort, or case-control;

(4) valid data odds ratios (OR) and 95% confidence intervals (CI) were provided, or raw data that could be transformed;

(5) literature quality rating > 5 and describe adjustment for potential confounders. The specific adjustment for confounding factors includes comorbidities (such as hypertension, diabetes, cardiovascular disease), lifestyle factors (physical activity levels, smoking status, alcohol consumption), socioeconomic status (education level, income), and demographic factors (age, gender, etc.).

Exclusion criteria for the study were as follows:

(1) duplicate literature;

(2) review and systematic evaluation of literature;

(3) non-Chinese and English literature.

### 2.3 Literature screening and extraction

2 researchers read the titles and abstracts of the studies for initial screening and read the remaining literature in its entirety to determine the final inclusion of the literature. 2 researchers used a pre-designed data extraction form to extract the basic information of the included literature, including the authors of the included studies, year of publication, baseline study year, country, type of study, participants/cases, age, gender (female %), and study content. The entire process of literature screening and extraction was done independently by 2 researchers, and disputes were resolved through discussion in case of disagreement. For studies with incomplete data, the corresponding author contacted the person in charge, and the study was deleted if valid data were still unavailable.

### 2.4 Literature evaluation

The quality of the included case-control and cohort studies was evaluated using the Newcastle Ottawa Scale (NOS) (Stang, 2010), which has a total score of 9, and a literature quality assessment of < 6 is considered to be a low-quality study. For the included cross-sectional studies, quality evaluation was performed using the evaluation tool recommended by the Agency for Healthcare Research and Quality (AHRQ) (Liang et al., 2022); the total score of this scale was 11, and the quality of the literature < 6 was classified as a low-quality study. Low-quality studies were excluded from this study.

### 2.5 Statistical analysis

Meta-analysis was performed by log-transforming the study-specific estimates. The heterogeneity of studies was characterized by Q-test and *I*^2^. Heterogeneity was small when *P* > 0.1 and *I*^2^ ≤ 50%, and a fixed-effects model was used for the combined analysis; statistical heterogeneity existed when *P* < 0.1 or *I*^2^ > 50% (Vu et al., 2021). When study heterogeneity was large, the source of study heterogeneity was found by article-by-article culling, or subgroup analysis was performed to reduce study heterogeneity, and if study heterogeneity still existed, a random-effects model was selected. We searched for sources of heterogeneity in studies of the relevance of visual impairment, hearing impairment, and dual sensory impairment to dementia in older adults through article-by-article exclusion. We conducted subgroup analyses to determine whether the correlation between visual impairment and hearing impairment and cognitive impairment in older adults differed in results among important variables, and we stratified our analyses primarily on the type of study, country, and sample size of included studies. Sensitivity analysis was applied to determine the stability of the study results; funnel plot and Egger’s test were used to determine whether the study had publication bias (Shang et al., 2021; Vu et al., 2021), and the difference was considered statistically significant at *P* < 0.05. The data analysis software utilized in this study was Reviewer Manager 5.4 and Stata 17.0.

## 3 Results

### 3.1 Search results

**FIGURE 1:**
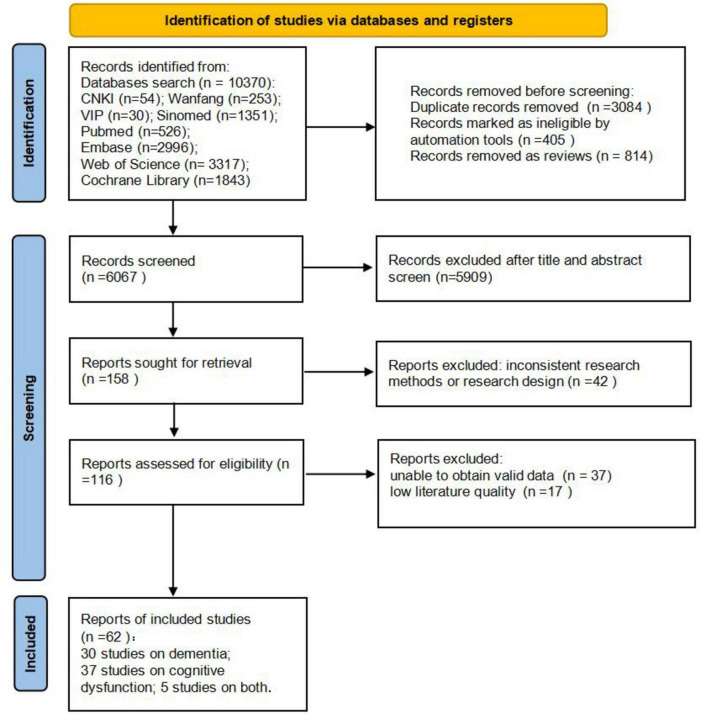
Literature screening process diagram.

A total of 10,370 studies were retrieved for this study, and we browsed the titles and abstracts of all studies. A total of 4,303 studies that did not meet the inclusion criteria were excluded; 5,909 studies that did not match the content of the study were excluded. Read the remaining 158 full-text exclusions: 42 studies with inconsistent study design, 37 studies with no access to valid data, and 17 studies with low-quality literature. Finally, 62 studies were included. The specific literature screening results are shown in Figure 1.

### 3.2 Basic characteristics and quality evaluation of literature

A total of 62 studies were included, 30 studies on dementia (Rogers and Langa, 2010; Gurgel et al., 2014; Hung et al., 2015; Deal et al., 2017; Golub et al., 2017; Amieva et al., 2018; Luo et al., 2018; Brenowitz et al., 2019; Davidson and Guthrie, 2019; Naël et al., 2019; Vassilaki et al., 2019; Hwang et al., 2020, 2022; Lee et al., 2020; Maruta et al., 2020; Tran et al., 2020; Brewster et al., 2021; Byeon et al., 2021; Chen et al., 2021; Ehrlich et al., 2021; Kuo et al., 2021; Pabst et al., 2021; Littlejohns et al., 2022; Mohammed et al., 2022; Stevenson et al., 2022; Huang et al., 2023; Killeen et al., 2023; Myrstad et al., 2023; Yu et al., 2023; Park et al., 2024), 37 studies on cognitive impairment (Rogers and Langa, 2010; Mitoku et al., 2016; Chen et al., 2017; Soto-Perez-de-Celis et al., 2018; Curhan et al., 2019; Davidson and Guthrie, 2019; Vassilaki et al., 2019; Curhan et al., 2020; Ma et al., 2020; Sardone et al., 2020; Cao et al., 2021; Chen, 2021; Ehrlich et al., 2021; Fang et al., 2021; Fenwick et al., 2021; Lee et al., 2021; Saji et al., 2021; Bikbov et al., 2022; Fuller-Thomson et al., 2022; Guthrie et al., 2022; Muhammad et al., 2022; Tai et al., 2022; Tomida et al., 2022; Vu et al., 2022; Wang et al., 2022; Dai et al., 2023; Li et al., 2023; Paiva et al., 2023; Xu et al., 2023, 2024; Yu et al., 2023; Jeong and Chang, 2024; Luo et al., 2024; Marmamula et al., 2024; Qi et al., 2024; Qin et al., 2024; Yang et al., 2024), and 5 studies reported on both dementia and cognitive impairment (Rogers and Langa, 2010; Davidson and Guthrie, 2019; Vassilaki et al., 2019; Ehrlich et al., 2021; Yu et al., 2023). Among the dementia studies, there were 25 cohort studies (Rogers and Langa, 2010; Gurgel et al., 2014; Deal et al., 2017; Golub et al., 2017; Amieva et al., 2018; Brenowitz et al., 2019; Davidson and Guthrie, 2019; Naël et al., 2019; Vassilaki et al., 2019; Hwang et al., 2020, 2022; Lee et al., 2020; Maruta et al., 2020; Tran et al., 2020; Brewster et al., 2021; Byeon et al., 2021; Chen et al., 2021; Ehrlich et al., 2021; Kuo et al., 2021; Pabst et al., 2021; Littlejohns et al., 2022; Mohammed et al., 2022; Stevenson et al., 2022; Myrstad et al., 2023; Park et al., 2024), 4 cross-sectional studies (Luo et al., 2018; Huang et al., 2023; Killeen et al., 2023; Yu et al., 2023), and 1 case-control study (Hung et al., 2015); 7 on dual sensory impairments, 15 on visual impairments, and 17 on hearing impairments; all had literature quality scores of ≥ 6, with a maximum of 11 (Huang et al., 2023). Among the cognitive dysfunction studies, there were 21 cohort studies (Rogers and Langa, 2010; Mitoku et al., 2016; Soto-Perez-de-Celis et al., 2018; Curhan et al., 2019, 2020; Davidson and Guthrie, 2019; Vassilaki et al., 2019; Ma et al., 2020; Cao et al., 2021; Chen, 2021; Ehrlich et al., 2021; Fang et al., 2021; Fuller-Thomson et al., 2022; Guthrie et al., 2022; Tai et al., 2022; Tomida et al., 2022; Vu et al., 2022; Dai et al., 2023; Xu et al., 2023; Qin et al., 2024; Yang et al., 2024), 16 cross-sectional studies (Chen et al., 2017; Sardone et al., 2020; Fenwick et al., 2021; Lee et al., 2021; Saji et al., 2021; Bikbov et al., 2022; Muhammad et al., 2022; Wang et al., 2022; Li et al., 2023; Paiva et al., 2023; Yu et al., 2023; Jeong and Chang, 2024; Luo et al., 2024; Marmamula et al., 2024; Qi et al., 2024; Xu et al., 2024); 8 on dual sensory impairment, 20 on visual impairment, and 20 on hearing impairment; all had a literature quality score of ≥ 6, with a maximum of 10 (Chen et al., 2017). The basic characteristics and quality evaluation of the literature are shown in Tables 1, 2.

**TABLE 1:** Basic characteristics of studies related to sensory disorders and dementia.

References	Baseline study year	Country	Study type	Participants/cases	Age (years)	Gender (female %)	Research contents	Quality evaluation
Byeon et al., 2021	2012–2014	Korea	Cohort study	6,520/201	≥ 60	56.87	Double sensory disorder	8
Davidson and Guthrie, 2019	2009–2014	Canada	Cohort study	352,656/69,213	≥ 65	63.2	Double sensory disorder	6
Hwang et al., 2022	1992–1999	America	Cohort study	2,927/307	≥ 65	58.2	Double sensory disorder; visual impairment; hearing impairment	9
Pabst et al., 2021	1997–2017	Germany	Cohort study	3,497/902	≥ 75	67.2	Visual impairment; hearing impairment	8
Mohammed et al., 2022	2003–2018	America	Cohort study	280/89	79.5 (5.2)	63	Hearing impairment	8
Deal et al., 2017	1999–2006	America	Cohort study	1,889/229	70–79	53	Hearing impairment	8
Gurgel et al., 2014	1995–2008	America	Cohort study	4,463/137	≥ 65	56.98	Hearing impairment	7
Brenowitz et al., 2019	1998–2014	America	Cohort study	1,810/336	70–79	51.82	Visual impairment; hearing impairment	8
Chen et al., 2021	2011–2018	America	Cohort study	10,676/ 2,371	≥ 65	59	Visual impairment;	9
Hwang et al., 2020	2000–2008	America	Cohort study	2,051/321	≥ 75	44.12	Double sensory disorder; visual impairment	7
Brewster et al., 2021	2005–2017	America	Cohort study	2,051/163	≥ 60	53.33	Hearing impairment	7
Rogers and Langa, 2010	1992–2005	America	Cohort study	625/168	≥ 71	61.6	Visual impairment;	8
Lee et al., 2020	2005–2011	China	Cohort study	15,576/1,349	≥ 65	63.8	Visual impairment;	7
Littlejohns et al., 2022	2006–2011	Britain	Cohort study	7,337/517	≥ 60	53.9	Visual impairment;	7
Amieva et al., 2018	1988–2014	France	Cohort study	3,588/876	≥ 65	57.8	Hearing impairment	8
Maruta et al., 2020	2010–2017	Japan	Cohort study	2,190/1,153	≥ 65	79.4	Double sensory disorder	6
Naël et al., 2019	2001–2012	France	Cohort study	7,736/882	≥ 65	61.3	Visual impairment	8
Kuo et al., 2021	2011–2020	America	Cohort study	7,562/4,234	≥ 65	58.3	Double sensory disorder; visual impairment; hearing impairment	8
Tran et al., 2020	2000–2002	America	Cohort study	1,061/42	66–84	100	Visual impairment	8
Stevenson et al., 2022	2009–2019	Britain	Cohort study	82,039/1,285	≥ 60	52.1	Hearing impairment	9
Park et al., 2024	2005–2019	Korea	Cohort study	44,728/1,875	≥ 60	44.1	Hearing impairment	7
Myrstad et al., 2023	2017–2019	Norway	Cohort study	7,135/1,089	≥ 70	55.3	Hearing impairment	8
Golub et al., 2017	1992–2012	America	Cohort study	1,881/56	76 (6.3)	70	Hearing impairment	8
Vassilaki et al., 2019	2004–2013	America	Cohort study	4,812/273	73.7 (9.6)	48.5	Hearing impairment	8
Ehrlich et al., 2021	2001–2008	America	Cohort study	351/97	≥ 70	55	Visual impairment;	7
Hung et al., 2015	1998–2011	China	Case-control	2,440/488	≥ 65	55.74	Hearing impairment	7
Yu et al., 2023	2021	China	Cross-sectional	1,120/103	≥ 60	59.29	Visual impairment;	7
Luo et al., 2018		China	Cross-sectional	250,752/5,277	≥ 65	52.5	Double sensory disorder; visual impairment; hearing impairment	8
Huang et al., 2023	2021	America	Cross-sectional	2,413/332	≥ 70	53.89	Hearing impairment	11
Killeen et al., 2023	2021	America	Cross-sectional	3,817/497	≥ 71	55.3	Visual impairment	9

**TABLE 2:** Basic characteristics of studies related to sensory impairment and cognitive impairment.

References	Baseline study year	Country	Study type	Participants/cases	Age (years)	Gender (female %)	Research contents	Quality evaluation
Davidson and Guthrie, 2019	2009–2014	Canada	Cohort study	352,656/69,213	≥ 65	63.2	Double sensory disorder	6
Soto-Perez-de-Celis et al., 2018	–	America	Cohort study	750/45	≥ 65	44	Double sensory disorder	8
Tomida et al., 2022	2013	Japan	Cohort study	4,471/1,052	≥ 70	52.3	Double sensory disorder; visual impairment;	6
Mitoku et al., 2016	2003–2009	Japan	Cohort study	1,754/360	≥ 65	65.51	Double sensory disorder; visual impairment; hearing impairment	7
Ehrlich et al., 2021	2001–2008	America	Cohort study	351 /97	≥ 70	55	Visual impairment;	7
Yang et al., 2024	2008–2018	China	Cohort study	6,862/1,712	≥ 65	51.2	Double sensory disorder; visual impairment; hearing impairment	8
Guthrie et al., 2022	2009–2014	Canada	Cohort study	106,920/13,609	≥ 65	68.4	Double sensory disorder; visual impairment; hearing impairment	6
Fuller-Thomson et al., 2022	2008–2017	Canada	Cohort study	5,405,135/2,599,869	≥ 65	56.4	Visual impairment;	6
Fang et al., 2021	2005–2012	China	Cohort study	105,208/4,542	75.23 ± 6.91	49.3	Visual impairment;	7
Chen et al., 2021	2011–2014	China	Cohort study	4,267/651	≥ 65	48.98	Hearing impairment	7
Cao et al., 2021	2002–2014	China	Cohort study	16,151/4,625	≥ 65	50.9	Visual impairment	8
Curhan et al., 2019	2008–2016	America	Cohort study	10,107/2,771	≥ 62	100	Hearing impairment	9
Curhan et al., 2020	2012–2014	America	Cohort study	20,193/5,106	≥ 66	100	Hearing impairment	9
Vu et al., 2022	2010–2016	Singapore	Cohort study	2,324/248	≥ 60	48.45	Visual impairment	8
Rogers and Langa, 2010	1992–2005	America	Cohort study	625/168	≥ 71	61.6	Visual impairment	8
Tai et al., 2022	1999–2011	China	Cohort study	4,208/3,861	≥ 60	48.1	Visual impairment;	7
Qin et al., 2024	2011–2012, 2014, and 2018	China	Cohort study	5,218/2,307	≥ 65	52.05	Hearing impairment	8
Ma et al., 2020	2014–2017	China	Cohort study	1,117/575	70–84	55.1	Hearing impairment	6
Dai et al., 2023	2018–2021	China	Cohort study	2,216/1,129	≥ 60	59.7	Visual impairment	6
Xu et al., 2023	2021	China	Cohort study	224/112	≥ 60	57.1	Hearing impairment	7
Vassilaki et al., 2019	2004–2013	America	Cohort study	4,812/273	73.7 (9.6)	48.5	Hearing impairment	8
Li et al., 2023	2022	China	Cross-sectional	2,242/1,006	≥ 60	54.5	Hearing impairment	8
Bikbov et al., 2022	2017–2020	Russia	Cross-sectional	731/242	≥ 85	72.5	Double sensory disorder; visual impairment; hearing impairment	7
Wang et al., 2022	2019	China	Cross-sectional	1,012/371	≥ 60	57.71	Hearing impairment	8
Fenwick et al., 2021	–	Singapore	Cross-sectional	874/281	≥ 60	51	Visual impairment	8
Paiva et al., 2023	2019	Brazil	Cross-sectional	1,335/274	≥ 60	63.7	Hearing impairment	6
Saji et al., 2021	2018	Japan	Cross-sectional	1,602/565	≥ 65	58.49	Hearing impairment	7
Chen et al., 2017	2011–2015	America	Cross-sectional	30,202/7,546	≥ 65	52	Visual impairment	10
Jeong and Chang, 2024	2020	Korea	Cross-sectional	9,692/3,858	≥ 65	55.2	Double sensory disorder; visual impairment; hearing impairment	7
Muhammad et al., 2022	2011	India	Cross-sectional	9,541/5,724	≥ 60	52.6	Visual impairment	6
Luo et al., 2024	2021	China	Cross-sectional	428/138	≥ 60	52.1	Visual impairment	6
Marmamula et al., 2024	–	India	Cross-sectional	965/260	≥ 60	63.4	Visual impairment	7
Qi et al., 2024	2019–2020	China	Cross-sectional	10,347/1,977	≥ 60	57.2	Hearing impairment	8
Lee et al., 2021	2009–2015	Korea	Cross-sectional	1,815,835/247,828	≥ 66	53.65	Hearing impairment	7
Sardone et al., 2020	2013–2018	Italy	Cross-sectional	1,647/260	≥ 65	55.07	Hearing impairment	7
Xu et al., 2024	2022	China	Cross-sectional	363/268	≥ 60	43.8	Hearing impairment	8
Yu et al., 2023	2021	China	Cross-sectional	1,120/103	≥ 60	59.29	Visual impairment	7

### 3.3 Meta-analysis of sensory impairment and dementia in older adults

#### 3.3.1 Dual sensory impairment

**FIGURE 2:**
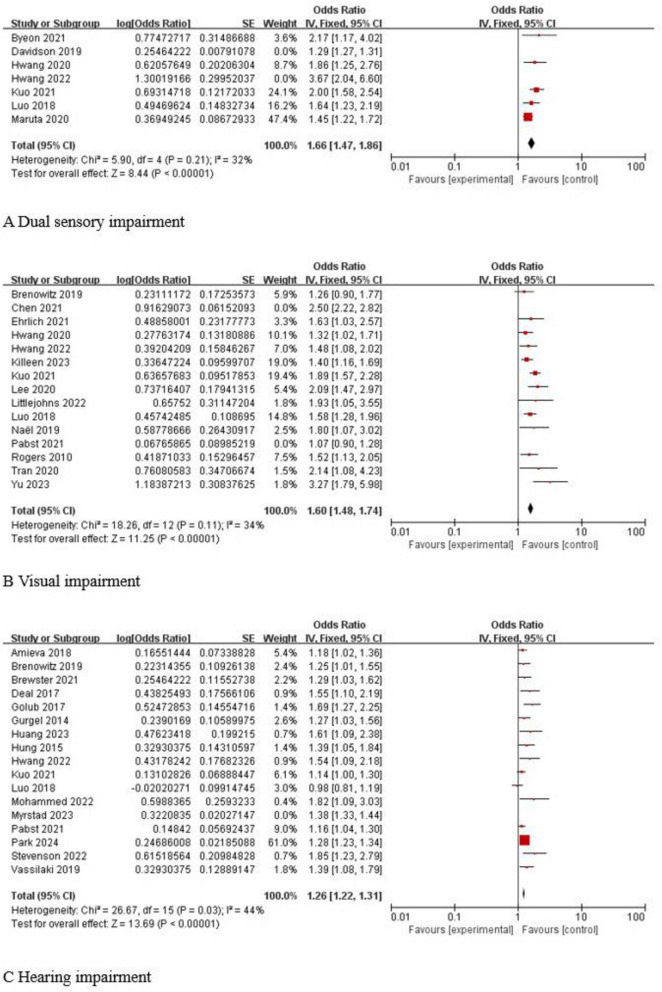
Forest plot of sensory impairment and dementia in older adults. **(A)** Dual sensory impairment and dementia. **(B)** Visual impairment and dementia. **(C)** Hearing impairment and dementia.

A total of seven studies (Luo et al., 2018; Davidson and Guthrie, 2019; Hwang et al., 2020, 2022; Maruta et al., 2020; Byeon et al., 2021; Kuo et al., 2021) reported the association between dual sensory impairment and dementia in older adults, and a test for heterogeneity found statistical heterogeneity across studies (*I*^2^ = 83%, *P* < 0.001). A sensitivity analysis was performed, revealing that the studies by Davidson and Guthrie (2019) and Hwang et al. (2022) were more heterogeneous. In Hwang et al.’s (2022), the log OR value reached a maximum of 1.30; in Davidson and Guthrie’s (2019), the log OR value reached a minimum of 0.25, while the log OR values in the remaining studies ranged from 0.37 to 0.77, and the heterogeneity was reduced after excluding two studies (*I*^2^ = 32%, *P* = 0.21), so a fixed-effects model was chosen for the combined analysis, and the results showed that dual sensory impairment was associated with dementia in older adults, and the results were statistically significant [OR 95% CI 1.66 (1.47, 1.86), *P* < 0.001]. As shown in Figure 2.

#### 3.3.2 Visual impairment

A total of 15 studies (Rogers and Langa, 2010; Luo et al., 2018; Brenowitz et al., 2019; Naël et al., 2019; Hwang et al., 2020, 2022; Lee et al., 2020; Tran et al., 2020; Chen et al., 2021; Ehrlich et al., 2021; Kuo et al., 2021; Pabst et al., 2021; Littlejohns et al., 2022; Killeen et al., 2023; Yu et al., 2023) reported the association between visual impairment and dementia in older adults, and a heterogeneity test found statistical heterogeneity among the studies (*I*^2^ = 84%, *P* < 0.001). A sensitivity analysis was performed and found that the studies of Chen et al. (2021) and Pabst et al. (2021) were more heterogeneous, and the heterogeneity was reduced by excluding these studies (*I*^2^ = 34%, *P* = 0.11), so a fixed-effects model was chosen for the combined analysis, and the results showed that visual impairment was associated with dementia in older adults, and the results were statistically significant [OR 95% CI 1.60 (1.48, 1.74), *P* < 0.001]. As shown in Figure 2.

#### 3.3.3 Hearing impairment

A total of 17 studies (Gurgel et al., 2014; Hung et al., 2015; Deal et al., 2017; Golub et al., 2017; Amieva et al., 2018; Luo et al., 2018; Brenowitz et al., 2019; Vassilaki et al., 2019; Brewster et al., 2021; Kuo et al., 2021; Pabst et al., 2021; Hwang et al., 2022; Mohammed et al., 2022; Stevenson et al., 2022; Huang et al., 2023; Myrstad et al., 2023; Park et al., 2024) reported the association between hearing impairment and dementia in older adults, and the heterogeneity test found that there was statistical heterogeneity among the studies (*I*^2^ = 58%, *P* < 0.001). A sensitivity analysis was performed and found that the study of Myrstad et al. (2023) was more heterogeneous, and the heterogeneity was reduced by excluding this study (*I*^2^ = 44%, *P* = 0.03), so a fixed-effects model was chosen for the combined analysis, and the results showed that hearing impairment was associated with dementia in older adults, and the results were statistically significant [OR 95% CI 1.26 (1.22, 1.31), *P* < 0.001]. As shown in Figure 2.

### 3.4 Meta-analysis of sensory impairment and cognitive impairment in older adults

#### 3.4.1 Dual sensory impairment

**FIGURE 3:**
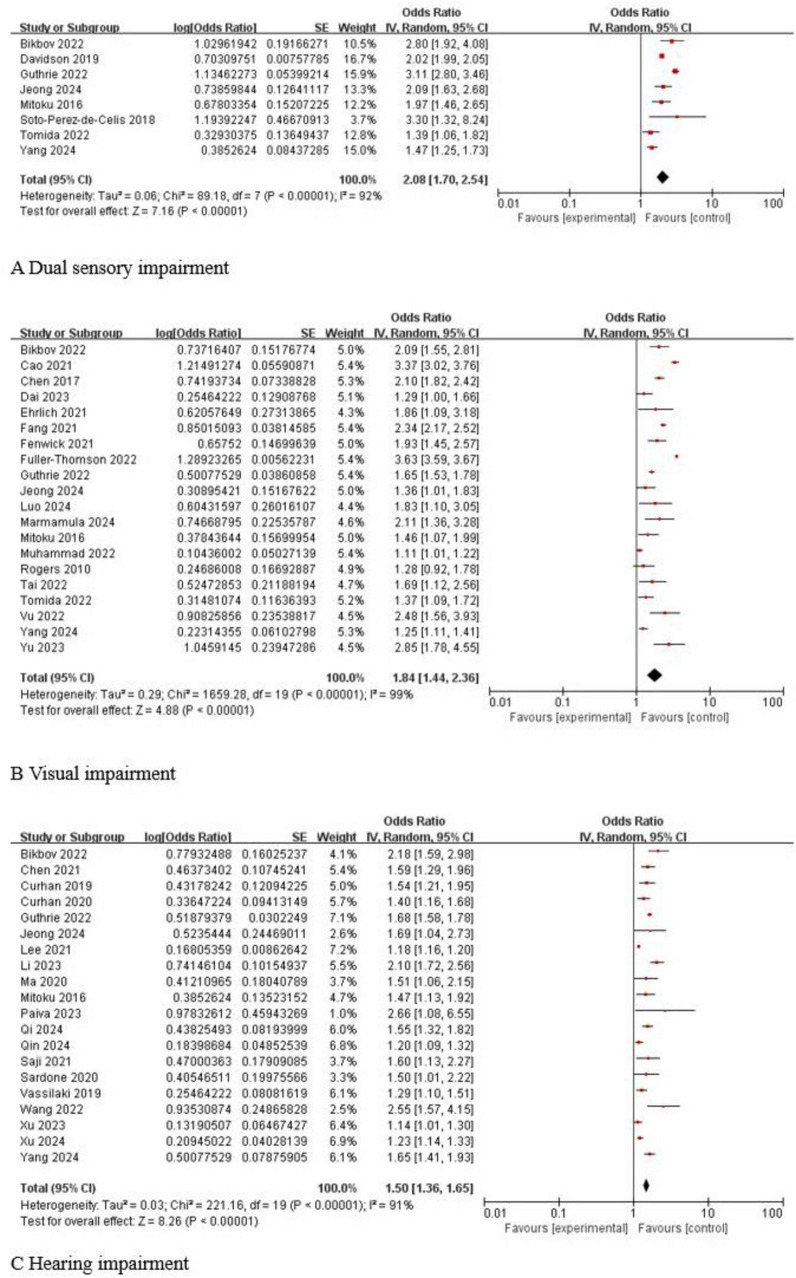
Forest plot of sensory impairment and cognitive impairment. **(A)** Dual sensory impairment and cognitive impairment. **(B)** Visual impairment and cognitive impairment. **(C)** Hearing impairment and cognitive impairment.

A total of 8 studies (Mitoku et al., 2016; Soto-Perez-de-Celis et al., 2018; Davidson and Guthrie, 2019; Bikbov et al., 2022; Guthrie et al., 2022; Tomida et al., 2022; Jeong and Chang, 2024; Yang et al., 2024) reported the association between dual sensory impairment and cognitive impairment in older adults, and the heterogeneity test found that there was statistical heterogeneity among the studies (*I*^2^ = 92%, *P* < 0.001). A sensitivity analysis was performed and found that the results of the studies were stable, so a random-effects model was chosen for data analysis, which showed that dual sensory impairment was associated with cognitive impairment in older adults, and the results were statistically significant [OR 95% CI 2.08 (1.70, 2.54), *P* < 0.001]. As shown in Figure 3.

#### 3.4.2 Visual impairment

A total of 20 studies (Rogers and Langa, 2010; Mitoku et al., 2016; Chen et al., 2017; Cao et al., 2021; Ehrlich et al., 2021; Fang et al., 2021; Fenwick et al., 2021; Bikbov et al., 2022; Fuller-Thomson et al., 2022; Guthrie et al., 2022; Muhammad et al., 2022; Tai et al., 2022; Tomida et al., 2022; Vu et al., 2022; Dai et al., 2023; Yu et al., 2023; Jeong and Chang, 2024; Luo et al., 2024; Marmamula et al., 2024; Yang et al., 2024) reported the association between visual impairment and cognitive impairment in older adults, and the heterogeneity test found that there was a statistically significant heterogeneity among the studies (*I*^2^ = 99%, *P* < 0.001). Sensitivity analysis was performed and found that the results of the studies were stable, so a random effects model was chosen for data analysis, which showed that visual impairment was associated with cognitive impairment in older adults and the results were statistically significant [OR 95% CI 1.84 (1.44, 2.36), *P* < 0.001]. As shown in Figure 3.

#### 3.4.3 Hearing impairment

A total of 20 studies (Mitoku et al., 2016; Curhan et al., 2019, 2020; Vassilaki et al., 2019; Ma et al., 2020; Sardone et al., 2020; Chen, 2021; Lee et al., 2021; Saji et al., 2021; Bikbov et al., 2022; Guthrie et al., 2022; Wang et al., 2022; Li et al., 2023; Paiva et al., 2023; Xu et al., 2023, 2024; Jeong and Chang, 2024; Qi et al., 2024; Qin et al., 2024; Yang et al., 2024) reported the association between hearing impairment and cognitive impairment in older adults, and the heterogeneity test found that there was statistically significant heterogeneity among the studies (*I*^2^ = 91%, *P* < 0.001). Sensitivity analysis was performed and found that the results of the studies were stable, so the random effects model was chosen for data analysis, and the results showed that hearing impairment was associated with cognitive impairment in older adults, and the results were statistically significant [OR 95% CI 1.50 (1.36, 1.65), *P* < 0.001]. As shown in Figure 3.

### 3.5 Subgroup analysis

#### 3.5.1 Visual impairment and cognitive impairment

A total of 8 cross-sectional studies reported the association between visual impairment and cognitive impairment in older adults. Meta-analysis results showed that visual impairment was associated with cognitive impairment in older adults and the results were statistically significant [OR 95% CI 1.82 (1.38, 2.40), *P* < 0.001] (Supplementary Figure 1). A total of 12 cohort studies reported the association between visual impairment and cognitive impairment in older adults. Meta-analysis showed that visual impairment was associated with cognitive impairment in older adults and the results were statistically significant [OR 95% CI 1.85 (1.39, 2.47), *P* < 0.001] (Supplementary Figure 1). A total of 7 studies from China reported the association between visual impairment and cognitive impairment in older adults. Meta-analysis showed that visual impairment was associated with cognitive impairment in older adults and the results were statistically significant [OR 95% CI 1.96 (1.41, 2.73), *P* < 0.001] (Supplementary Figure 2). A total of 13 studies from other countries reported the association between visual impairment and cognitive impairment in older adults. Meta-analysis showed that visual impairment was associated with cognitive impairment in older adults and the results were statistically significant [OR 95% CI 1.79 (1.26, 2.53), *P* < 0.05] (Supplementary Figure 2). A total of 15 studies (sample size < 10,000) reported the association between visual impairment and cognitive impairment in old age. Meta-analysis results showed that visual impairment was associated with cognitive impairment in old age and the results were statistically significant [OR 95% CI 1.58 (1.38, 1.81), *P* < 0.001] (Supplementary Figure 3). A total of 5 studies (sample size > 10,000) reported the association between visual impairment and cognitive impairment in old age. Meta-analysis showed that visual impairment was associated with cognitive impairment in old age and the results were statistically significant [OR 95% CI 2.51 (1.76, 3.57), *P* < 0.001] (Supplementary Figure 3).

#### 3.5.2 Hearing impairment and cognitive impairment

A total of 10 cross-sectional studies reported the association between hearing impairment and cognitive impairment in old age. Meta-analysis results showed that hearing impairment was associated with cognitive impairment in old age and the results were statistically significant [OR 95% CI 1.61 (1.39, 1.87), *P* < 0.001] (Supplementary Figure 4). A total of 10 cohort studies reported the association between hearing impairment and cognitive impairment in old age. Meta-analysis results showed that hearing impairment was associated with cognitive impairment in old age and the results were statistically significant [OR 95% CI 1.43 (1.27, 1.60), *P* < 0.001] (Supplementary Figure 4). A total of 9 studies from China reported the association between hearing impairment and cognitive impairment in old age. Meta-analysis showed that hearing impairment was associated with cognitive impairment in old age and the results were statistically significant [OR 95% CI 1.49 (1.30, 1.71), *P* < 0.001] (Supplementary Figure 5). A total of 11 studies from other countries reported the association between hearing impairment and cognitive impairment in old age. Meta-analysis showed that hearing impairment was associated with cognitive impairment in old age and the results were statistically significant [OR 95% CI 1.53 (1.31, 1.79), *P* < 0.001] (Supplementary Figure 5). A total of 15 studies (sample size < 10,000) reported the association between hearing impairment and cognitive impairment in older adults. Meta-analysis showed that hearing impairment was associated with cognitive impairment in older adults and the results were statistically significant [OR 95% CI 1.52 (1.36, 1.71), *P* < 0.001] (Supplementary Figure 6). A total of 5 studies (sample size > 10,000) reported the association between hearing impairment and cognitive impairment in older adults. Meta-analysis showed that hearing impairment was associated with cognitive impairment in older adults and the results were statistically significant [OR 95% CI 1.46 (1.18, 1.79), *P* < 0.05] (Supplementary Figure 6).

### 3.6 Publication bias

In dementia-related studies, the funnel plot of visual impairment with dementia in older adults was symmetric (Egger test: *P* = 0.395) (Supplementary Figure 7); and the funnel plot of hearing impairment with dementia in older adults was symmetric (Egger test: *P* = 0.910) (Supplementary Figure 8). In studies of cognitive impairment, the funnel plot of visual impairment versus cognitive impairment in old age was slightly asymmetric (Egger test: *P* = 0.00) (Supplementary Figure 9); the funnel plot of hearing impairment versus cognitive impairment in old age was slightly asymmetric (Egger test: *P* = 0.002) (Supplementary Figure 10).

## 4 Discussion

This study provides a referable basis for reducing the occurrence of dementia and cognitive impairment in older adults. This study not only assessed the correlation of visual impairment and hearing impairment with dementia and cognitive impairment in older adults but also extended the theoretical basis by systematically analyzing the correlation of dual sensory impairment with dementia and cognitive impairment in older adults. Therefore, it is recommended that older adults with sensory impairments undergo routine screening, particularly in primary healthcare settings, with regular cognitive assessments (e.g., using the Montreal Cognitive Assessment) to detect early signs of dementia or cognitive decline. Provide timely intervention measures, such as hearing aids, vision correction equipment, or sensory rehabilitation training, while regularly conducting relevant themed health education activities to encourage older adults to actively manage their vision and hearing problems actively, thereby reducing the incidence of cognitive impairment. The results of this meta-analysis suggest that visual impairment, hearing impairment, and dual sensory impairment can predict dementia and cognitive impairment in older adults.

Visual impairment and hearing impairment are now increasingly recognized as potentially modifiable factors for dementia in older adults. Studies have reported that the risk of dementia in older adults with visual impairment is 8 times higher than in older adults without visual impairment (Kuo et al., 2021). The Lancet Dementia Council, in a study on dementia prevention, care, and intervention, showed that hearing impairment is the most significant cause of dementia in older adults (Livingston et al., 2020). Dual sensory impairment is an extremely vulnerable subgroup that has the highest prevalence in the older adult population (Hwang et al., 2022), and it is difficult to compensate for by sensory substitution compared to single sensory impairments (e.g., compensating for dysfunction due to visual impairment through the auditory system). Therefore, older adults with dual sensory impairment are at higher risk for dementia (Kuo et al., 2021). Research has shown that visual impairment and hearing impairment can explain their relationship with dementia through several mechanisms. Sensory impairment can lead to depression, social isolation, reduced physical activity, and functional limitations, all of which increase the risk of dementia (Hwang et al., 2020, 2022; Kuo et al., 2021). Research indicates that social support and mental health interventions play a crucial role in mitigating the adverse effects of sensory impairments on dementia risk (Hwang et al., 2020, 2022; Kuo et al., 2021). Social support acts as a protective factor: robust social networks can mitigate social isolation stemming from sensory deficits, promote regular physical and mental activities, and reduce the incidence of dementia and cognitive impairment. For instance, group-based interventions such as cognitive stimulation programs provide structured social interaction and mental engagement, compensating for diminished sensory input and reducing feelings of isolation. Regarding mental health interventions, targeted treatment for depression in individuals with sensory impairments (e.g., cognitive behavioral therapy) can disrupt the “sensory deficit-depression-dementia” cascade. Furthermore, integrating mental health screening into routine care for older adults with vision or hearing loss enables early identification and management of psychological distress, thereby reducing the incidence of cognitive impairment. In addition, sensory deficits lead to reduced activation of central sensory pathways, which in turn leads to structural and functional changes in the brain, such as atrophy of frontal brain regions induced by afferent nerve blockade, which puts pressure on brain circuits (Hwang et al., 2020, 2022; Kuo et al., 2021). At the same time, sensory impairments limit the neural resources required for optimal performance of cognitive tasks by increasing cognitive load (Hwang et al., 2020; Kuo et al., 2021). In addition, hearing impairment and visual impairment are associated with vascular lesions, such as white matter high signaling and microangiopathy, which are important contributors to dementia (Hwang et al., 2020). The higher risk of dementia in patients with dual sensory impairment may be that individuals compensate for the functional limitations of single sensory impairment through the undamaged sensory system (Hwang et al., 2020; Kuo et al., 2021; Shang et al., 2021). The common cause hypothesis proposes that the association between sensory impairment and dementia reflects common pathological processes such as microvascular lesions and inflammation (Hwang et al., 2020, 2022; Kuo et al., 2021). However, this needs to be validated by more relevant mechanisms.

Currently, studies have shown that the risk of cognitive impairment in older adults with hearing impairment is 2.66 times higher than in those without hearing impairment (Paiva et al., 2023). A meta-analysis indicated (Shang et al., 2021) that the risk of developing cognitive impairment was 35% higher in older adults with visual impairment than in those without visual impairment. In addition, a 10-year cohort study, which was analyzed after adjusting for the effects of confounders such as education, BMI, smoking, alcohol consumption, marital status, and chronic diseases on outcomes, showed that older adults with dual sensory impairments had a higher risk of developing cognitive dysfunction than those with single sensory impairments (Yang et al., 2024). The association between patients with sensory disorders and cognitive impairment may stem from changes in brain structure and function (Yang et al., 2024). Studies have shown that patients with sensory impairments have reduced gray matter density and reduced temporal lobe volume in the brain, and these cortical changes and reductions may contribute to cognitive decline (Shang et al., 2021; Vu et al., 2021; Yang et al., 2024). Sensory impairments also limit the interaction of older adults in their environments, for example, older adults affected by visual impairments and hearing impairments largely do not participate in physical and social activities, which indirectly contributes to an increased risk of cognitive impairment in older adults (Shang et al., 2021; Vu et al., 2021). In addition, studies have shown that sensory impairment and cognitive impairment share many common risk factors (e.g., age, smoking, hypertension, diabetes, etc.) (Xu et al., 2024; Yang et al., 2024) and pathological underpinnings (e.g., β-amyloid deposition) (Shang et al., 2021; Vu et al., 2021; Yang et al., 2024). β-amyloid deposition, a common histopathological feature of the brains of cognitively impaired patients, is also present in the retinal pigment epithelium of patients with visual impairment and β-amyloid deposition leads to mitochondrial dysfunction, inflammation, and vascular regulation, which further explains the association between sensory impairment and cognitive impairment (Byeon et al., 2021; Shang et al., 2021; Vu et al., 2021). The association between sensory impairment and cognitive impairment currently lacks a definitive mechanism. Future research should focus on conducting more high-quality longitudinal studies, such as time-series and causal studies, to determine the directional relationship between sensory impairment and cognitive impairment. Mediating effect analyses should be conducted to identify modifiable mediating factors (e.g., social engagement, physical activity) and moderating factors (e.g., age) influencing this association, thereby providing a reference basis for targeted intervention measures.

This meta-analysis gives us some insights. First, we found that visual impairment, hearing impairment, and dual sensory impairment were predictive of dementia and cognitive impairment in older adults through systematic and comprehensive assessment. Sensory impairment is potentially modifiable and provides a practical reference for reducing the occurrence of dementia and cognitive impairment in older adults. Second, given that dual sensory impairment is an important risk factor for the development of dementia and cognitive impairment, it is recommended that public health and healthcare should focus on this factor in the development of interventions and focus on screening this population. Finally, this meta-analysis was conducted strictly with the PRISMA reporting statement, and the larger sample size and higher quality of the included literature may inform future studies in similar settings.

Similarly, this meta-analysis has some limitations. First, there was a high level of heterogeneity among the included studies, possibly due to differences in sample size, measurement tools, and follow-up time. Therefore, more studies could be included in the future to reduce the source of heterogeneity. Second, this study included a limited number of studies related to dual sensory impairment with dementia and cognitive impairment in older adults, which may affect the comprehensiveness of the analyzed results. Finally, some of the studies included in our analysis were cross-sectional in design, precluding the establishment of causal relationships. This means that early subclinical cognitive decline itself may lead to visual and hearing impairments, rather than merely being a consequence of sensory deficits causing cognitive impairment. Moreover, cross-sectional designs cannot account for temporal sequences. Therefore, more prospective and large-scale cohort studies are needed in future research, which can longitudinally track the occurrence and progression of sensory and cognitive impairments, clarify directional relationships, and strengthen causal inference.

## 5 Conclusion

Overall, our study suggests that visual impairment, hearing impairment, and dual sensory impairment are potential risk factors for dementia and cognitive impairment in older adults. Future research requires more high-quality longitudinal studies to explore the relationship between sensory impairments and dementia and cognitive impairment, with a particular focus on the following specific directions. First, conduct separate studies on different types of sensory impairments (such as mild versus severe visual/hearing impairments, single versus dual sensory impairments) to determine whether their predictive effects on dementia and cognitive impairment in older adults differ. Second, investigate potential mechanisms, such as how sensory impairments contribute to cognitive decline through pathways involving brain structure and function (e.g., cortical atrophy, neurotransmitter changes), psychosocial factors (e.g., social isolation, depression), or vascular pathology. Additionally, high-quality randomized controlled trials should be conducted to validate whether interventions targeting sensory impairments (e.g., hearing aid use, visual rehabilitation training) can effectively delay or prevent the onset of dementia and cognitive impairment in older adults, thereby providing more precise guidance for clinical practice and public health strategies.

## Data Availability

The original contributions presented in this study are included in this article/Supplementary material, further inquiries can be directed to the corresponding author.
