# Supplementary material for: The validity of visual and hearing impairment in predicting dementia and cognitive impairment in older adults: a systematic review and meta-analysis
Source: Front Aging Neurosci. 2026 Jan 16;17:1656686. doi: 10.3389/fnagi.2025.1656686 (PMC12856923; doi:10.3389/fnagi.2025.1656686)
Supplement: Supplementary file 1 [file Data_Sheet_1.docx]

**Supplementary materials**

**Search strategy**

1. **Pubmed (526)**

#1 "Vision Disorders"[MeSH Terms]

#2 'Vision Disorder':ab,ti OR 'Visual Disorders':ab,ti OR 'Disorders, Visual':ab,ti OR 'Disorder, Visual':ab,ti OR 'Visual Disorder':ab,ti OR 'Macropsia':ab,ti OR 'Macropsias':ab,ti OR 'Hemeralopia':ab,ti OR 'Hemeralopias':ab,ti OR 'blindness ':ab,ti OR 'Micropsia':ab,ti OR 'Micropsias':ab,ti OR 'Vision Disability':ab,ti OR 'Disabilities, Vision':ab,ti OR 'Disability, Vision':ab,ti OR 'Vision Disabilities':ab,ti OR 'Visual Impairment':ab,ti OR 'Impairments, Visual':ab,ti OR 'Impairment, Visual':ab,ti OR 'Visual Impairments':ab,ti OR 'Metamorphopsia':ab,ti OR 'Metamorphopsias ':ab,ti OR 'visual acuity ':ab,ti OR 'vision acuity ':ab,ti OR 'visual loss ':ab,ti

#3 "Hearing Loss"[MeSH Terms]

#4 'Loss, Hearing':ab,ti OR 'Hearing Impairment':ab,ti OR 'Hypoacusis':ab,ti OR 'Hypoacuses':ab,ti OR 'Deafness, Transitory':ab,ti OR 'Deafnesses, Transitory':ab,ti OR 'Transitory Deafness':ab,ti OR 'Transitory Deafnesses':ab,ti OR 'Hearing Loss, Transitory':ab,ti OR 'Loss, Transitory Hearing':ab,ti OR 'Transitory Hearing Losses':ab,ti OR 'Transitory Hearing Loss ':ab,ti OR 'Hearing acuity ':ab,ti OR 'Hearing disorders ':ab,ti OR 'Hearing difficulty':ab,ti

#5 "dual sensory loss"[Title/Abstract] OR "dual sensory impairment"[Title/Abstract] OR "dual impairments"[Title/Abstract] OR "sensory impairments"[Title/Abstract]

#6 #1 OR #2 OR #3 OR #4 OR #5

#7 "older*"[Title/Abstract] OR "elder*"[Title/Abstract] OR "senior*"[Title/Abstract] OR "geriatric*"[Title/Abstract] OR "aged"[Title/Abstract]

#8 "Cognitive Dysfunction"[MeSH Terms]

#9 'Cognitive Dysfunctions':ab,ti OR 'Dysfunction, Cognitive':ab,ti OR 'Dysfunctions, Cognitive':ab,ti OR 'Cognitive Disorder':ab,ti OR 'Cognitive Disorders':ab,ti OR 'Disorder, Cognitive':ab,ti OR 'Disorders, Cognitive':ab,ti OR 'Cognitive Impairments':ab,ti OR 'Cognitive Impairment':ab,ti OR 'Impairment, Cognitive':ab,ti OR 'Impairments, Cognitive ':ab,ti OR 'Cognitive Decline':ab,ti OR 'Cognitive Declines':ab,ti OR 'Decline, Cognitive':ab,ti OR 'Declines, Cognitive ':ab,ti OR 'Cognitive Function ':ab,ti
#10 "Dementia"[MeSH Terms]

#11 "Dementias"[Title/Abstract] OR "Amentia"[Title/Abstract] OR "Amentias"[Title/Abstract]

#12 #8 OR #9 OR #10 OR #11

#13 #6 AND #7 AND #12

**2 Web of Science (3317)**

#1 ((((((((((((((((TS=("visual acuity")) OR TS=("vision acuity")) OR TS=("visual impairment")) OR TS=("vision impairment")) OR TS=("visual loss")) OR TS=("vision loss")) OR TS=("partial sight")) OR TS=(blindness)) OR TS=("Hearing acuity")) OR TS=("Hearing Loss")) OR TS=("Hearing impairment")) OR TS=("Hearing disorders")) OR TS=("Hearing difficulty")) OR TS=("Dual Sensory Loss")) OR TS=("Dual Sensory Impairment")) OR TS=("Dual impairments")) OR TS=("Sensory Impairments")

#2 ((((TS=(older*)) OR TS=(elder*)) OR TS=(senior*)) OR TS=(geriatric*)) OR TS=(aged)

#3 ((((((((((TS=("Cognitive Dysfunction")) OR TS=("Cognitive Dysfunctions")) OR TS=("Dysfunction, Cognitive")) OR TS=("Dysfunctions, Cognitive")) OR TS=("Cognitive Disorder")) OR TS=("Cognitive Disorders")) OR TS=("Cognitive Impairments")) OR TS=("Cognitive Impairment")) OR TS=("Cognitive Decline")) OR TS=("Cognitive Declines")) OR TS=("Cognitive Function")

#4 ((((TS=(Dementia)) OR TS=(Dementias)) OR TS=(Amentia)) OR TS=(Amentias)) OR TS=(alzheimer*)

#5 #3 OR #4

#6 #1 AND #2 AND #5

1. **Embase (2996)**

#1 'visual acuity'/exp OR 'visual acuity' OR (visual AND ('acuity'/exp OR acuity)) OR 'vision acuity':ab,ti OR 'visual impairment':ab,ti OR 'vision impairment':ab,ti OR 'visual loss':ab,ti OR 'vision loss':ab,ti OR 'partial sight':ab,ti OR blindness:ab,ti OR 'hearing acuity':ab,ti OR 'hearing loss':ab,ti OR 'hearing impairment':ab,ti OR 'hearing disorders':ab,ti OR 'hearing difficulty':ab,ti OR 'dual sensory loss':ab,ti OR 'dual sensory impairment':ab,ti OR 'dual impairments':ab,ti OR 'sensory impairments':ab,ti

#2 older* OR elder*:ab,ti OR senior*:ab,ti OR geriatric*:ab,ti OR aged:ab,ti

#3 'cognitive dysfunction'/exp OR 'cognitive dysfunction' OR (cognitive AND dysfunction) OR 'cognitive dysfunctions':ab,ti OR 'dysfunction, cognitive':ab,ti OR 'dysfunctions, cognitive':ab,ti OR 'cognitive disorder':ab,ti OR 'cognitive disorders':ab,ti OR 'cognitive impairments':ab,ti OR 'cognitive impairment':ab,ti OR 'cognitive decline':ab,ti OR 'cognitive declines':ab,ti OR 'cognitive function':ab,ti

#4 'dementia'/exp OR dementia OR dementias:ab,ti OR amentia:ab,ti OR amentias:ab,ti OR alzheimer*:ab,ti

#5 #3 OR #4

#6#1 AND #2 AND #5

1. **Cochrane (1843)**

#1 (visual acuity):ti,ab,kw OR (vision impairment):ti,ab,kw OR (visual loss):ti,ab,kw OR (vision acuity):ti,ab,kw OR (vision impairment):ti,ab,kw

#2 (Hearing acuity):ti,ab,kw OR (Hearing Loss):ti,ab,kw OR (Hearing impairment):ti,ab,kw OR (Hearing disorders):ti,ab,kw OR (Hearing difficulty):ti,ab,kw

#3 (Dual Sensory Loss):ti,ab,kw OR (Dual Sensory Impairment):ti,ab,kw OR (Dual impairments):ti,ab,kw OR (Sensory Impairments):ti,ab,kw

#4 #1 OR #2 OR #3

#5 (older*):ti,ab,kw OR (elder*):ti,ab,kw OR (senior*):ti,ab,kw OR (geriatric*):ti,ab,kw OR (aged):ti,ab,kw

#6 (Cognitive Declines):ti,ab,kw OR (Cognitive Dysfunctions):ti,ab,kw OR (Cognitive Function):ti,ab,kw OR (Cognitive Disorders):ti,ab,kw OR (Cognitive Declines)

#7(Dementia):ti,ab,kw OR (Dementias):ti,ab,kw OR (Amentia):ti,ab,kw OR (Amentias):ti,ab,kw OR (alzheimer*):ti,ab,kw

#8 #6 OR #7

#9 #4 AND #5 AND #8

**5知网（54）**

#1视觉障碍 + 视力下降 + 视力受损 + 听觉障碍 + 听力受损 + 听力下降 + 双重感觉障碍 + 双重感觉丧失 + 感觉障碍 + 感觉丧失

#2老人 + 老年人

#3认知障碍 + 认知能力下降 + 认知能力减退 + 认知功能 + 痴呆 + 阿尔茨海默症

#4 #1 AND #2 AND #3

**6万方（253）**

#1视觉障碍 OR 视力下降 OR 视力受损 OR 听觉障碍 OR 听力受损 OR 听力下降 OR 双重感觉障碍 OR 双重感觉丧失 OR 感觉障碍 OR 感觉丧失

#2老人 OR 老年人

#3认知障碍 OR 认知能力下降 OR 认知能力减退 OR 认知功能 OR 痴呆 OR 阿尔茨海默症

#4 #1 AND #2 AND #3

**7维普（30）**

#1视觉障碍 OR 视力下降 OR 视力受损 OR 听觉障碍 OR 听力受损 OR 听力下降 OR 双重感觉障碍 OR 双重感觉丧失 OR 感觉障碍 OR 感觉丧失

#2老人 OR 老年人

#3认知障碍 OR 认知能力下降 OR 认知能力减退 OR 认知功能 OR 痴呆 OR 阿尔茨海默症

#4 #1 AND #2 AND #3

1. **Sinomed (1351)**

#1 "视觉障碍"[不加权:扩展]

#2 "听觉障碍"[不加权:扩展]

#3 "视觉障碍"[常用字段:智能] OR "视力下降"[常用字段:智能] OR "视力受损"[常用字段:智能] OR "听觉障碍"[常用字段:智能] OR "听力受损"[常用字段:智能] OR "听力下降"[常用字段:智能] OR "双重感觉障碍"[常用字段:智能] OR "双重感觉丧失"[常用字段:智能] OR "感觉障碍"[常用字段:智能]

#4 #1 OR #2 OR #3

#5 "老人"[常用字段:智能] OR "老年人"[常用字段:智能]

#6 "认知障碍"[常用字段:智能] OR "认知能力下降"[常用字段:智能] OR "认知能力减退"[常用字段:智能] OR "认知功能"[常用字段:智能] OR "痴呆"[常用字段:智能] OR "阿尔茨海默症"[常用字段:智能]

#7 #4 AND #5 AND #6

**Supplemental Figures**


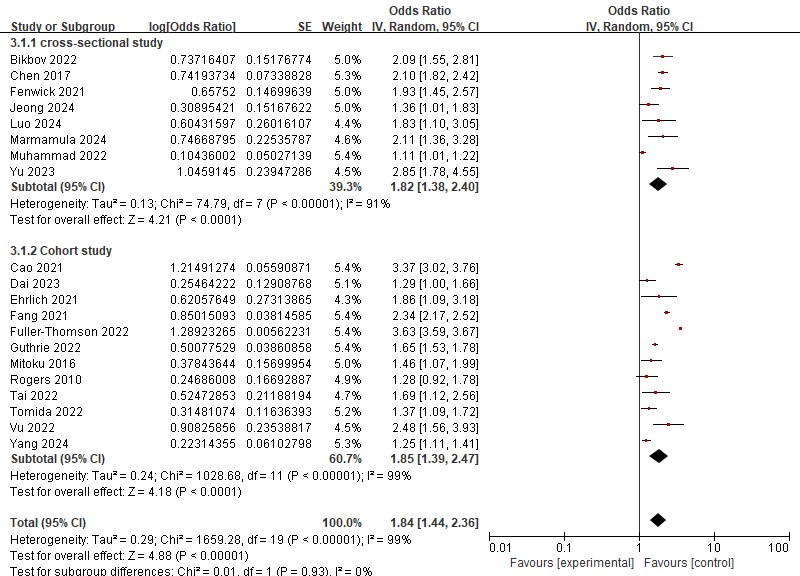


Figure S1 Visual impairment and cognitive impairment in older adults (study type)


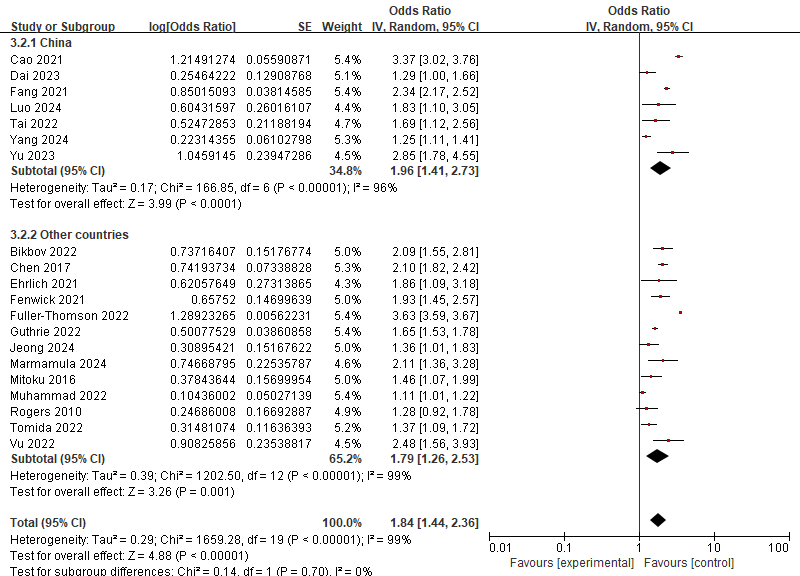


Figure S2 Visual impairment and cognitive impairment in older adults (country)


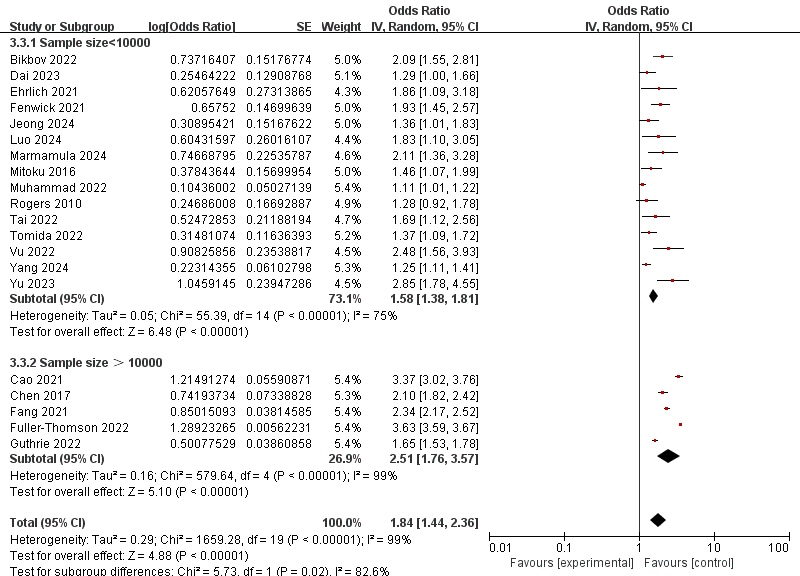


Figure S3 Visual impairment and cognitive impairment in older adults (sample size)


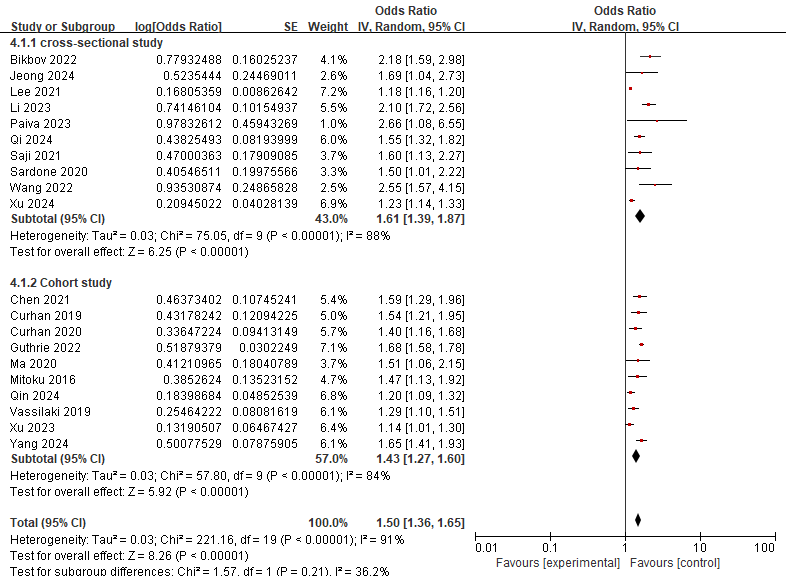


Figure S4 Hearing impairment and cognitive impairment in older adults (study type)


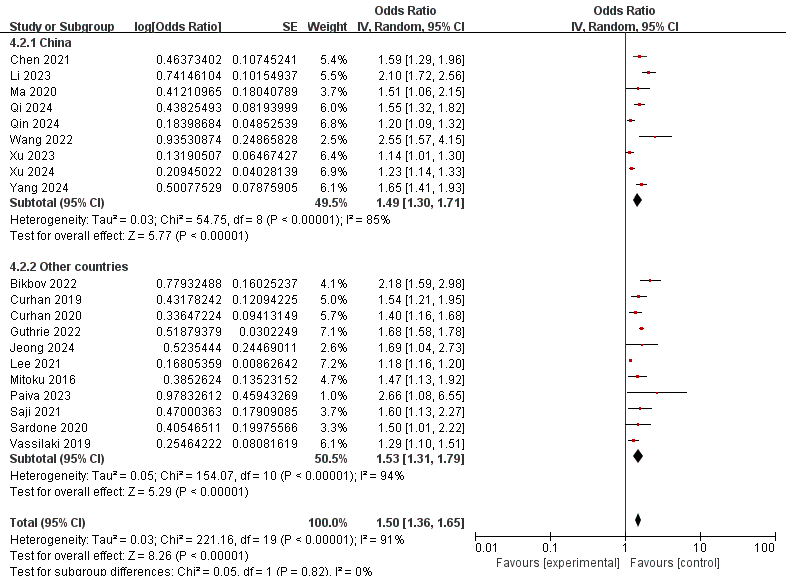


Figure S5 Hearing impairment and cognitive impairment in older adults (country)


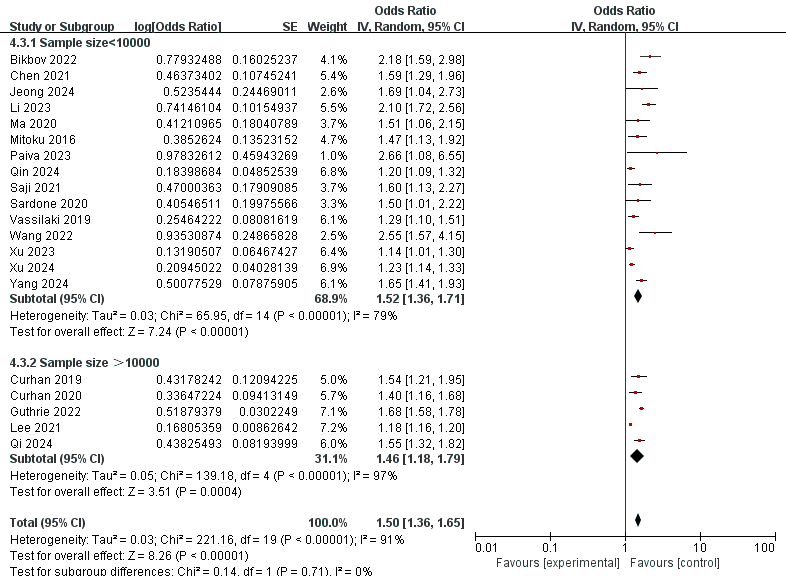


Figure S6 Hearing impairment and cognitive impairment in older adults (sample size)

Egger test: *P* = 0.395


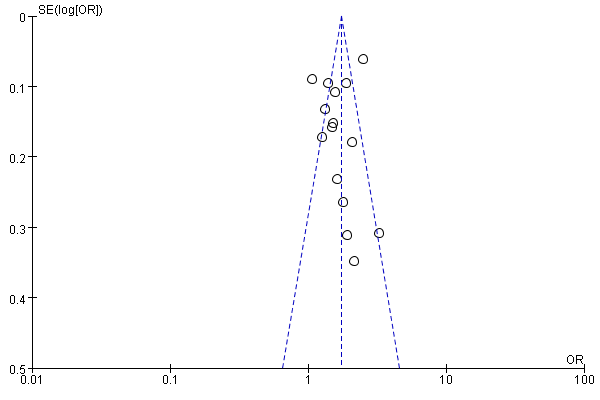


Figure S7 Funnel plot of visual impairment and dementia in older adults

Egger test: *P* = 0.910

Figure S8 Funnel plot of hearing impairment and dementia in older adults

Egger test: *P* = 0.00

Figure S9 Funnel plot of visual impairment and cognitive impairment in older adults

Egger test: *P* = 0.002

Figure S10 Funnel plot of hearing impairment and cognitive impairment in older adults
